# Lumican, an Exerkine, Protects against Skeletal Muscle Loss

**DOI:** 10.3390/ijms231710031

**Published:** 2022-09-02

**Authors:** Han Jin Cho, Young-Sun Lee, Da Ae Kim, Sung Ah Moon, Seung Eun Lee, Seung Hun Lee, Jung-Min Koh

**Affiliations:** 1 Asan Institute for Life Sciences, Asan Medical Center, Seoul 05505, Korea; 2 Virus Facility, Research Animal Resource Center, Korea Institute of Science and Technology, Seoul 02792, Korea; 3 Division of Endocrinology and Metabolism, Asan Medical Center, University of Ulsan College of Medicine, Seoul 05505, Korea

**Keywords:** lumican, exerkine, muscle loss, myogenesis, integrin

## Abstract

Exerkines are soluble factors secreted by exercised muscles, mimicking the effects of exercise in various organs, including the muscle itself. Lumican is reportedly secreted from muscles; however, its roles in skeletal muscle remain unknown. Herein, we found that lumican mRNA expression in the extensor digitorum longus was significantly higher in exercised mice than in unloading mice, and lumican stimulated myogenesis in vitro. Additionally, lumican knockdown significantly decreased muscle mass and cross-sectional area (CSA) of the muscle fiber in the gastrocnemius muscle of exercised mice. Lumican upregulated phosphorylation of p38 mitogen-activated protein kinase (MAPK) and a p38 inhibitor near completely blocked lumican-stimulated myogenesis. Inhibitors for integrin α2β1 and integrin ανβ3 also prevented lumican-stimulated myogenesis. Systemic lumican treatment, administered via the tail vein for 4 weeks, significantly increased relative muscle masses by 36.1% in ovariectomized mice. In addition, intramuscular lumican injection into unloaded muscles for 2 weeks significantly increased muscle mass by 8.5%. Both intravenous and intramuscular lumican treatment significantly increased muscle CSA. Our in vitro and in vivo experiments indicate that lumican is a muscle-secreted exerkine that affords protection against muscle loss by activating p38 MAPK via integrin receptors.

## 1. Introduction

Sarcopenia is characterized by the progressive and general loss of the skeletal muscle and strength [1]. It can be attributed to multiple causes, including aging, lack of exercise, and sex hormone deficiency [2]. As the aging population rapidly increases worldwide, sarcopenia is emerging as an important challenge in aging societies. However, no antisarcopenic drug has been approved, and a novel therapeutic target for sarcopenia needs to be identified.

Skeletal muscle is a musculoskeletal organ that allows movement and protects internal organs. Recently, skeletal muscle was recognized as an endocrine organ, as it secretes soluble factors, namely myokines, that participate in inter-organ crosstalk with other organs/tissues [3]. Importantly, it has been proposed that exercise-induced myokines, called exerkines, can modulate the multi-systemic benefits of exercise [4]. To date, several exerkine molecules, such as irisin, apelin, and myonectin, have been discovered [5,6,7,8], and efforts to identify new exerkines are ongoing, given that these molecules could be novel therapeutic targets mediating the beneficial health effects of exercise [9]. Interestingly, irisin, as an exerkine, was shown to exert pro-myogenic effects [10], suggesting that exerkines may afford beneficial effects on skeletal muscle in an autocrine manner.

Lumican, a member of the family of small leucine-rich proteoglycans, was originally discovered in chicken cornea [11]. Lumican deficiency reportedly causes corneal opacity and skin fragility in mice [12,13]. Subsequently, lumican was identified as a key regulator in several cellular processes, such as wound healing [14,15], angiogenesis [16], cell migration [17], and adhesion [18]. More recently, we have reported that lumican secreted from muscle cells could stimulate bone formation [19], consistent with the concept that lumican may exhibit a myokine-like role [20]. However, to the best of our knowledge, there is no current report regarding the role of lumican in skeletal muscle. In the present study, we performed in vitro and in vivo experimental experiments to generate evidence demonstrating that lumican could promote myoblast differentiation as an exerkine, thus affording a novel therapeutic target against sarcopenia.

## 2. Results

### 2.1. Exercise Stimulates Lumican Expression in Skeletal Muscles

**Figure 1:**
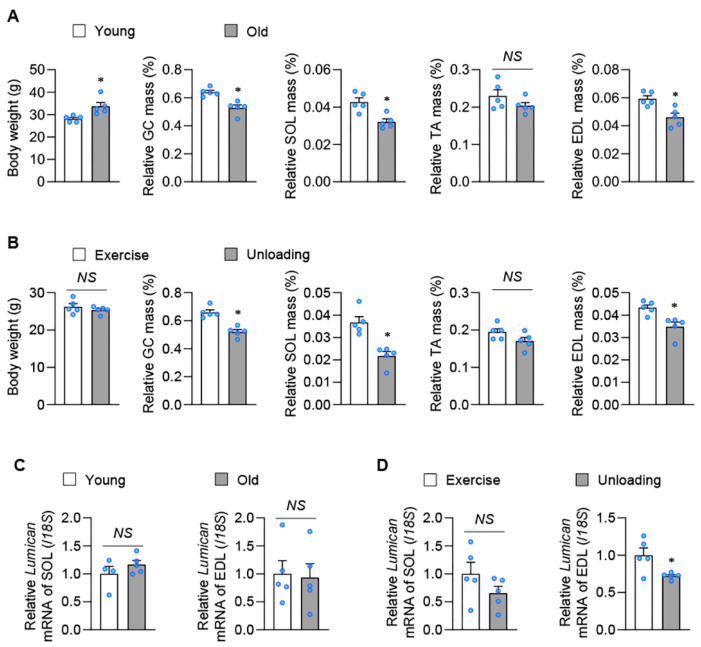
Lumican expression in skeletal muscles in various mouse models. (**A**,**B**) Body composition parameters, including body weight and muscle mass, were determined in young and old mice (n = 5 per group) (**A**) and in exercised and unloading mice (n = 5 per group). (**B**). Experimental mouse models are described in the Materials and Methods. Relative muscle mass is expressed as the percentage of body weight. (**C**,**D**) Expression levels of lumican mRNA were measured by real-time PCR in muscles of young and old mice (**C**) and exercised and unloading mice (**D**). Each bar represents the mean ± standard error of the mean (SEM). * *p* < 0.05 between the two groups. *NS*, not significant. GC, gastrocnemius. SOL, soleus. TA, tibialis anterior. EDL, extensor digitorum longus.

Skeletal muscle atrophy is typically attributed to aging and a lack of exercise; thus, expression levels of lumican were examined in skeletal muscles of mouse models corresponding to these two factors. We observed that the relative muscle mass was significantly decreased in most skeletal muscles of old (Figure 1A) and unloading (Figure 1B) mice when compared with young and exercised mice, respectively. Expression levels of lumican mRNA were determined in soleus (SOL) and extensor digitorum longus (EDL), given that SOL and EDL primarily consist of type 1 and type 2 muscle fibers, respectively. Both muscles exhibited comparable lumican expression between old and young mice (Figure 1C). In addition, the difference in lumican expression was also insignificant between SOL of exercised and unloading mice (Figure 1D). However, mRNA expression was significantly higher in exercised EDL (39.0 ± 13.6%) than in unloading EDL. These findings suggested that lumican could be a muscle-secreted exerkine, especially from type II muscle fibers.

### 2.2. Lumican Stimulates Myogenesis In Vitro

**Figure 2:**
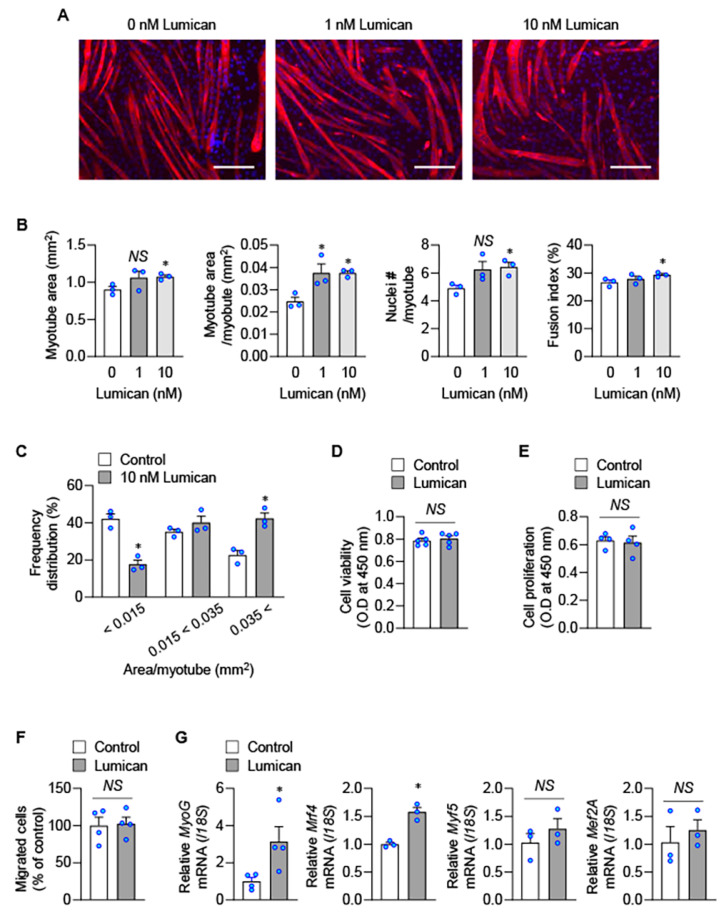
Lumican-mediated myoblast differentiation. (**A**–**C**) C2C12 cells were differentiated with indicated lumican concentrations for 3 days. Myotubes were stained with the anti-myosin heavy chain antibody (**A**), and morphological parameters of myotubes, such as total myotube area, myotube area per myotube, nuclei # per myotube, and fusion index, were quantified (**B**). Frequency distribution of myotubes was also determined (**C**). (**D**,**E**) C2C12 cells were treated with or without 10 nM lumican for 24 h, and cell viability (**D**) and proliferation (**E**) were examined using Cell counting kit-8 and Cell Proliferation ELISA, respectively. (**F**) Migration of C2C12 cells was examined using a type IV collagen-coated filter with 10 nM lumican. Migrated cells were quantitated. (**G**) C2C12 cells were incubated with or without 10 nM lumican for 24 h, and real-time PCR was performed. Each bar represents the mean ± standard error of the mean (SEM). Scale bars: 200 μm. * *p* < 0.05 vs. the untreated control. *NS*, not significant.

To examine the effect of lumican on myogenesis, C2C12 murine myoblasts were treated with recombinant lumican, and we observed that lumican stimulated myogenesis in a dose-dependent manner (Figure 2A). Especially, 10 nM lumican significantly increased all studied parameters associated with in vitro myogenesis, including total myotube area, myotube area per myotube, nuclei number per myotube, and fusion index (Figure 2B). Lumican also increased the number of large myotubes, while decreasing the number of small myotubes (Figure 2C). Lumican acted similarly on cells at various stages of myogenic differentiation from the beginning of differentiation (Supplementary Figure S1).

**Figure 3:**
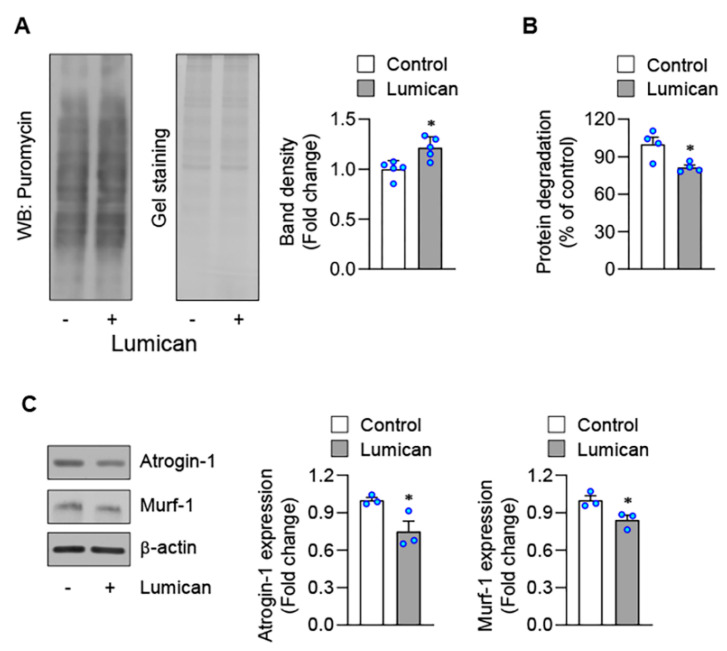
Lumican-mediated protein metabolism. (**A**) Protein synthesis was determined by detecting puromycin-labeled peptides. C2C12 cells were treated with 10 nM lumican for 30 min and lysed after 30 min of incubation with puromycin. Cell lysates were analyzed by western blotting with an antibody against puromycin. EZ-Gel staining was performed to assess equal amounts of protein in the sodium dodecyl sulfate-polyacrylamide gel electrophoresis wells. Quantitative results are shown in the right panel. (**B**) Protein degradation was measured in C2C12 myoblasts labeled with [^14^C]-L-leucine for 18 h, and then treated for 9 h with 10 nM lumican. TCA-soluble radioactivity released in the medium was measured, and protein degradation was calculated as described in the Materials and Methods. (**C**) C2C12 cells were differentiated for 24 h. Then, cells were incubated in serum-free DMEM for 1 h to induce protein degradation. Cells were treated with or without 10 nM lumican in serum-free DMEM for 9 h. Total cell lysates were subjected to western blotting with their relevant antibodies. Quantitative results are shown in the right panel. Each bar represents the mean ± standard error of the mean (SEM). * *p* < 0.05 vs. the untreated control. DMEM, Dulbecco’s Modified Eagle Medium; TCA, trichloroacetic acid; WB, western blotting.

Myogenesis is a multi-step process involving proliferation, migration, and differentiation of myoblasts [21]. We observed that lumican did not affect the number of viable cells (Figure 2D), proliferation (Figure 2E), and migration (Figure 2F) of C2C12 myoblasts; however, lumican could increase the mRNA expression levels of myogenic differentiation-related factors, such as myogenin and myogenic regulatory factor Mrf4 (Figure 2G). Muscular protein homeostasis also plays a critical role in skeletal muscle atrophy [22]. Lumican increased protein synthesis (Figure 3A) but decreased protein degradation (Figure 3B). Furthermore, lumican suppressed expression levels of muscle-specific E3 ubiquitin ligases, including atrogin-1 and Murf-1 (Figure 3C), critical factors associated with protein degradation [23]. Collectively, these findings indicated that lumican could stimulate myogenesis by enhancing differentiation and inducing the positive protein balance of myoblasts.

### 2.3. Lack of Lumican Contributes to Muscle Atrophy in Exercised Mice

**Figure 4:**
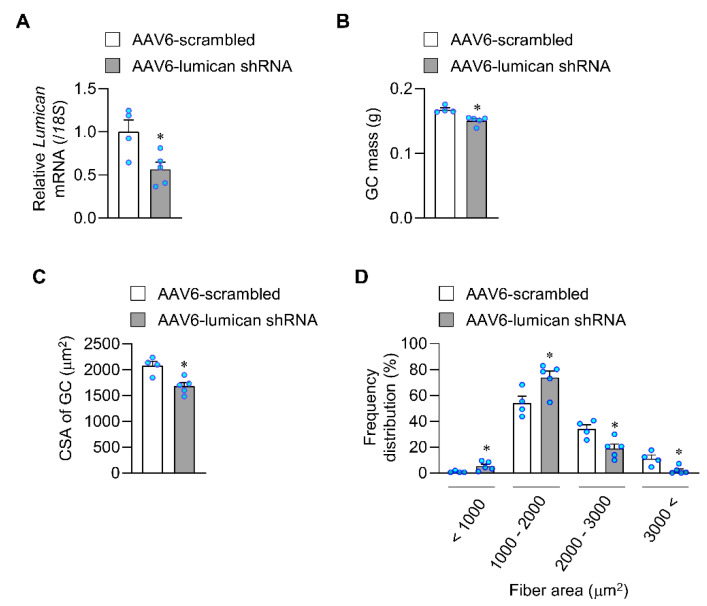
Muscle mass following intramuscular lumican knockdown in voluntarily exercised mice. Eight-week-old male C57BL/6 mice were administered AAV6-scrambled-mCherry (n = 4) or AAV6-lumican shRNA-mCherry (n = 5) into the GC muscle. The mice were sacrificed after 4 weeks, and GC muscles were harvested, weighed, and fixed in 4% paraformaldehyde. (**A**) Lumican mRNA expression in the GC muscle was determined using real-time PCR. (**B**,**C**) Muscle weights (**B**) and cross-sectional areas (CSAs, **C**) of the AAV6-infected GC muscle were quantitated. (**D**) Frequency distribution of the AAV6-infected fiber area was determined. Each bar represents the mean ± standard error of the mean (SEM). * *p* < 0.05 vs. the AAV6-scrambled group. GC, gastrocnemius.

We next examined the effect on lumican knockdown in skeletal muscles of exercised mice, given that exercise stimulated lumican expression. We injected adeno-associated-virus (AAV)6-lumican shRNA or AAV6-scrambled into gastrocnemius (GC) muscles (Figure 4A) and subsequently trained mice by performing voluntary wheel exercises for 4 weeks. AAV6 afforded partial infection, possibly due to the large size of GC muscles. However, AAV6-infected areas were similar between muscles treated with AAV6-scrambled (43.4 ± 8.2%) and AAV6-lumican shRNA (40.6 ± 6.4%) (Supplementary Figure S2), and lumican expression levels were significantly lower in GC muscles treated with AAV6-lumican shRNA than in those treated with AAV6-scrambled (Figure 4A). Interestingly, we noted that the muscle mass was significantly lower in muscles treated with AAV6-lumican shRNA than in those treated with AAV6-scrambled (Figure 4B), although we did not note any other signs of muscle atrophy, such as internalized nuclei, loss of nuclei, and loss of morphology (data not shown). In addition, we measured the cross-sectional area (CSA) of muscle fibers. Lumican knockdown significantly reduced the CSA of muscle fibers to 81.0 ± 3.3% when compared with the control group (Figure 4C), increased the number of small fibers and decreased that of large fibers (Figure 4D). Overall, these results indicated that the lack of lumican contributed to muscle atrophy in exercised mice.

### 2.4. Lumican Stimulates Myogenesis by Activating the p38 Mitogen-Activated Protein Kinase (MAPK) Signaling Pathway

**Figure 5:**
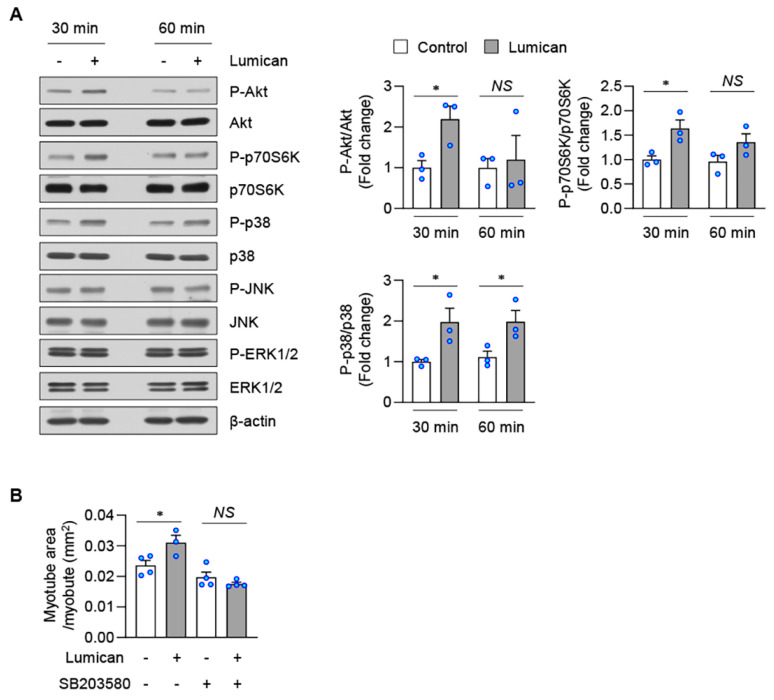
Lumican-stimulated myogenesis and protein balance mediated by p38 MAPK. (**A**) C2C12 cells were treated with or without 10 nM lumican for indicated periods. Total cell lysates were subjected to western blotting with relevant antibodies. Quantitative results are shown in the right panels. (**B**) C2C12 cells were differentiated with 10 nM lumican in the presence or absence of SB203580 for 3 days. Myotubes were stained with the anti-myosin heavy chain antibody. Quantitative results of myotube area per myotube are shown. Each bar represents the mean ± standard error of the mean (SEM). * *p* < 0.05 vs. lumican-untreated control. *NS*, not significant; MAPK, mitogen-activated protein kinase.

It has been reported that extracellular lumican activates Akt and MAPKs [15,19,24,25]. The activation of Akt and MAPKs can upregulate myogenesis [26,27,28]. Herein, we observed that lumican only transiently upregulated Akt phosphorylation in C2C12 cells (Figure 5A). Mammalian target of rapamycin (mTOR) is a downstream Akt target [26,29]. Lumican also transiently upregulated the phosphorylation level of p70S6K, a well-known downstream effector of mTOR [29] (Figure 5A). Conversely, lumican treatment consistently activated p38 MAPK in C2C12 cells (Figure 5A). In addition, pretreatment with a p38 inhibitor almost completely blocked lumican-stimulated myogenesis (Figure 5B, Supplementary Figure S3). Lumican treatment did not alter the phosphorylation of c-Jun N-terminal kinase (JNK) and extracellular signal-regulated kinase (ERK) (Figure 5A). These findings suggested that p38 MAPK activation, at least in part, could mediate the effects of lumican on muscle cells.

### 2.5. Integrins Are Putative Lumican Receptors in Muscle Cells

**Figure 6:**
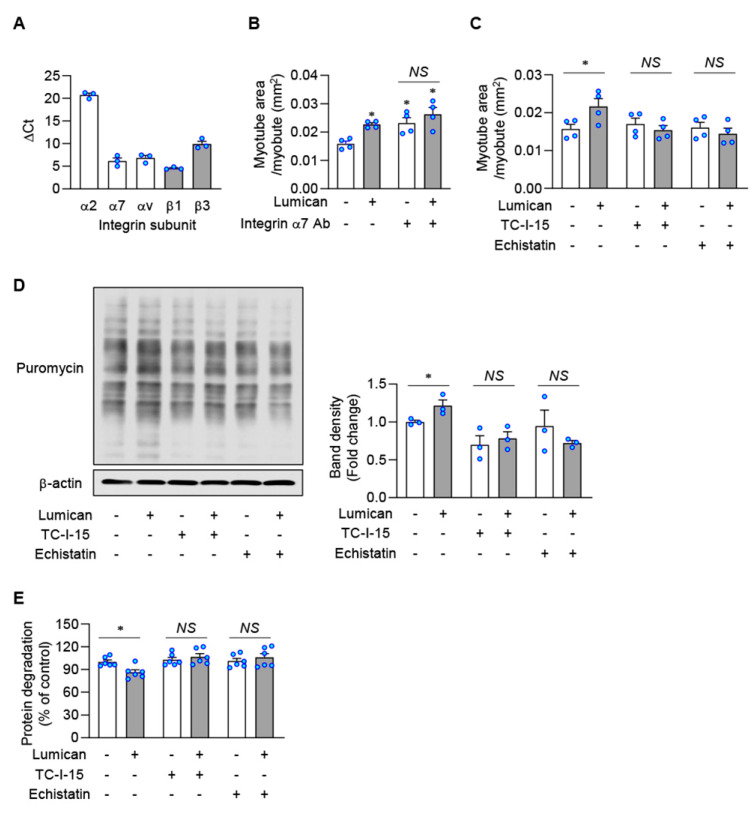
Lumican-stimulated myogenesis and protein balance mediated by integrins. (**A**) mRNA levels of integrin subunits in C2C12 cells were determined by real-time PCR. (**B**) C2C12 cells were differentiated with 10 nM lumican in the presence or absence of integrin α7 neutralizing antibody for 3 days. Myotubes were stained with the anti-myosin heavy chain antibody. Quantitative results of myotube area per myotube are shown. (**C**) C2C12 cells were differentiated with 10 nM lumican in the presence or absence of integrin inhibitors, such as TC-I-15 and echistatin, for 3 days. Quantitative results of myotube area per myotube are shown. (**D**) C2C12 cells were pre-treated with or without integrin inhibitors for 30 min. Then, cells were treated with 10 nM lumican for 30 min and underwent lysis after 30 min of incubation with puromycin. Protein synthesis was determined by detecting puromycin-labeled peptides by western blotting. Quantitative results are shown in the right panel. (**E**) Protein degradation was measured in C2C12 myoblasts cultured with lumican and/or integrin inhibitors, as described in Figure 3. Each bar represents the mean ± standard error of the mean (SEM). * *p* < 0.05 vs. lumican-untreated control. *NS*, not significant.

It is well-known that lumican interacts with integrins that function as its receptor [14,16,17,18,19]. We examined mRNA expression levels of several integrins in C2C12 cells, demonstrating that expression levels of α2, α7, αν, β1, and β3 were detectable, consistent with previous reports [30,31] (Figure 6A). Surprisingly, treatment with an integrin α7 blocking antibody rather stimulated in vitro myogenesis (Figure 6B, Supplementary Figure S4A), suggesting that integrin α7 might not be a lumican receptor. TC-I-15 and echistatin were assessed as inhibitors of integrin α2β1 and integrin ανβ3, respectively. Pretreatment with both inhibitors could almost completely block lumican-stimulated myogenesis (Figure 6C, Supplementary Figure S4B). Furthermore, pretreatment with both inhibitors could prevent lumican-stimulated protein synthesis (Figure 6D), as well as lumican-suppressed protein degradation (Figure 6E). Accordingly, integrins α2β1 and ανβ3 might be putative lumican receptors in muscle cells.

### 2.6. Lumican Treatment Increases Muscle Mass in Mouse Models Exhibiting Muscle Loss

**Figure 7:**
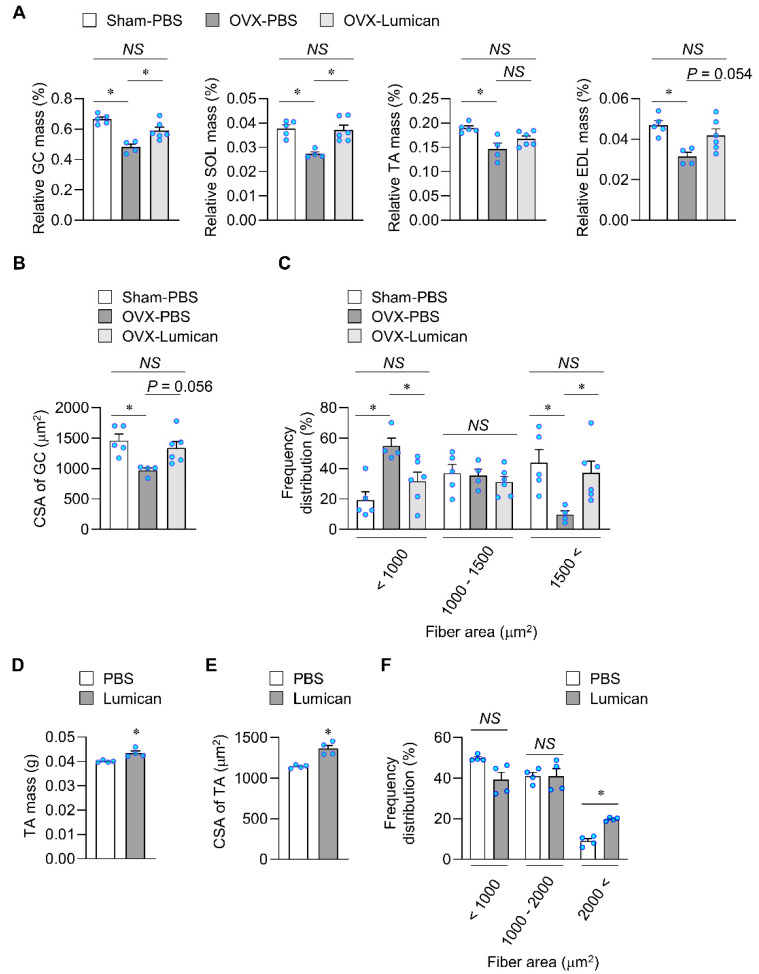
Systemic and intramuscular lumican treatment in mice exhibiting muscle loss. (**A**–**C**) Ovariectomized (OVX) mice were intravenously injected with PBS or 2 μg lumican for 4 weeks, as described in the Materials and Methods. The mice (n = 4–6 per group) were sacrificed, and relevant muscles were harvested, weighed, and fixed in 4% paraformaldehyde. Relative muscle mass is expressed as a percentage of body weight (**A**). GC muscle sections were stained with hematoxylin and eosin, and quantitative results of their cross-sectional areas (CSAs) are shown (**B**). Frequency distribution of the fiber area was also determined (**C**). (**D**–**F**) Male C57BL/6 mice (n = 4) were subjected to the tail suspension, and lumican (5 μg/mouse) was injected into the left TA muscle twice weekly for 2 weeks. The mice were sacrificed, and relevant muscles were harvested; muscle weights are shown (**D**). TA muscle sections were stained with hematoxylin and eosin, and quantitative results of their CSAs are shown (**E**). Frequency distribution of the fiber area was also determined (**F**). Each bar represents the mean ± standard error of the mean (SEM). * *p* < 0.05 between the two groups. *NS*, not significant. GC, gastrocnemius; SOL, soleus; TA, tibialis anterior; EDL, extensor digitorum longus; PBS, phosphate-buffered saline.

Sex hormone deficiency can cause skeletal muscle loss [32,33]. To examine the therapeutic effects of lumican, we performed bilateral ovariectomy (OVX) in female mice, subsequently injecting 2 μg lumican or phosphate-buffered saline (PBS) through the tail vein for 4 weeks. Compared with sham-operated mice, OVX mice exhibited a reduction in all examined relative muscle masses (Figure 7A). Lumican treatment significantly enhanced the relative muscle masses of GC and SOL by 22.7% and 36.1%, respectively, in OVX mice when compared with untreated OVX mice. Lumican treatment marginally increased the relative muscle mass of EDL by 33.1% (*p* = 0.054). We performed hematoxylin and eosin (H&E) staining to examine the CSA of GC muscles (Supplementary Figure S5A). The CSA of muscle fibers was reduced in OVX mice when compared with that of sham-operated mice, and lumican treatment marginally increased the CSA in OVX mice (*p* = 0.056) (Figure 7B). Notably, lumican treatment decreased the number of small fibers but increased that of large fibers in OVX mice (Figure 7C).

Next, hindlimb unloading was induced by tail suspension in mice, and lumican was injected intramuscularly into the tibialis anterior (TA) muscles for 2 weeks. Lumican treatment increased the muscle mass by 8.5 ± 4.3% (Figure 7D). In addition, lumican treatment increased TA muscle CSA (Figure 7E, Supplementary Figure S5B), and increased the number of large fibers (Figure 7F). Collectively, these results indicated that lumican treatment could restore muscle loss caused by sex hormone deficiency and unloading in vivo.

## 3. Discussion

We have previously reported that lumican secreted from muscle cells can stimulate bone formation in vitro, suggesting the role of lumican as a myokine exhibiting an anabolic action on bone [19]. In the present study, we, for the first time, reported experimental evidence demonstrating that lumican is a muscle-derived exerkine that could prevent muscle loss. Herein, we observed that exercise stimuli increased lumican expression in skeletal muscles, and lumican knockdown following treatment with relevant shRNAs decreased skeletal muscle mass in exercised mice. In addition, in vitro experiments revealed a potential underlying mechanism for lumican. Lumican increased myogenesis by stimulating muscle cell differentiation and inducing positive protein balance in muscle cells. Furthermore, lumican activated p38 MAPK to stimulate myogenesis. Integrins α2β1 and ανβ3 are considered putative lumican receptors in muscle cells. Finally, we revealed that both systemic and intramuscular administration of lumican induced muscle hypertrophy, thereby increasing muscle mass in mice with sex hormone deficiency- and disuse-induced loss in muscle mass. Thus, our findings provide novel insights clarifying the involvement of lumican in skeletal muscle biology as an exerkine and suggest that lumican could be developed as a therapeutic agent against muscle loss.

Our in vivo results clearly showed that lumican knockdown decreased muscle mass in exercised mice, despite the poor infection efficiency (40%) afforded by AAV6-lumican shRNA. Considering that exercise stimulated lumican expression, as shown in Figure 1D, we examined the effect of lumican knockdown on muscles under exercise which may maximize the knockdown’s effect. These findings suggest that lumican may be a muscle-derived exerkine that acts on muscles in an autocrine manner. Furthermore, lumican knockdown significantly decreased muscle fiber CSAs. Consistently, intramuscular and systemic lumican treatment also increased muscle fiber CSAs. Accordingly, lumican may induce muscle hypertrophy rather than hyperplasia. The in vitro experimental results support this interpretation, given that lumican stimulated muscle cell differentiation and induced positive protein balance but did not impact muscle cell proliferation.

In 2003, Perderson first proposed the concept of myokines, which were defined as cytokines and peptides produced and released by muscle fibers, exerting either autocrine, paracrine, or endocrine effects [34,35]. It has been reported that myokines facilitate communication between muscles and other organs, as well as within the muscle itself. Muscle-derived interleukin-6, first classified as a myokine, was reported to enhance lipolysis and fat oxidation in adipose tissue, enhance insulin-stimulated glucose uptake and glucose output from the liver, stimulate appetite, and promote muscle hypertrophy [35]. In particular, myokines can be subdivided into exerkines, which are increased following exercise. Irisin, apelin, and myonectin are well-known exerkines [5,6,7,8]. Irisin and apelin reportedly exert beneficial effects on the muscle itself [7,10], whereas the effect of myonectin on muscle remains unknown. Our results show that lumican is an additional exerkine affording beneficial effects on skeletal muscle. We noted that unloading decreased lumican expression in EDL but not in soleus (Figure 1D). EDL has relatively more type 2 muscle fibers than soleus [36], thus type 2 muscle fibers may mainly respond to stimuli to affect lumican expression. It was consistent to the previous report that expression of genes coding an extracellular matrix (ECM) like lumican was reportedly higher in EDL than SOL [36]. However, aging did not affect lumican expression in both muscles. We cannot precisely explain the reason because aging also causes muscle atrophy in type 2 fibers. Lumican expression may differentially respond to types of stimuli, or the response may be insensitive in aged skeletal muscles. These are necessary to be clarified in the future.

The role of the p38 MAPK pathway is well-established in myogenesis. Several studies have shown that phosphorylation and activity of p38 gradually increase with myoblast differentiation into myotubes [28,37,38]. Inhibition of p38 blocked myotube formation, but constitutive activation of p38 accelerated myogenic differentiation, clearly indicating that p38 is a positive regulator of myogenic differentiation. In addition, inhibition of p38 could decrease the rate of protein synthesis in C2C12 cells [26]. Activated p38 can mediate phosphorylation of S6K and 4E-BP1 [39,40], thereby promoting protein synthesis by activating ribosomal protein S6 and releasing the translation initiation factor eIF-4E, respectively. These results indicate that p38 signaling is also involved in regulating protein synthesis. These are consistent with our observations that lumican increases myotube formation and enhances protein synthesis by activating p38. In contrast, lumican only transiently stimulated the Akt-mTOR signaling axis. Thus, it seems unlikely that this axis may be pivotal for mediating the action of lumican in muscle cells.

Integrins are widely recognized as lumican receptors [14,16,17,18,19] and are heterodimeric proteins composed of α and β subunits. Numerous studies have shown that lumican-induced biological effects are mediated via integrin α2β1 [14,16,17,19], and that they directly bound [17,19]. Reportedly, integrins α4, α5, α6, α7, αν, and β1 are present in migrating myoblasts [30], and we detected the expression of α2 and β3. Although we did not examine all muscle-expressed integrins, we noted that inhibitors of α2β1 and ανβ3 prevented all lumican-induced changes in muscle cells, including stimulation of myogenesis and protein synthesis and suppression of protein degradation. Thus, these integrins, at least in part, might be putative lumican receptors.

Interestingly, intramuscular and systemic administration of lumican significantly protected against muscle loss in vivo, suggesting that lumican can be developed as a bio-drug against diseases associated with muscle loss, including sarcopenia. However, one of the main disadvantages of therapeutic peptides and proteins is their poor half-life. To the best of our knowledge, there are no previous reports evaluating the pharmacokinetics of lumican. Extracellular lumican is endocytosed by immune cells (macrophages and dendritic cells) [25], indicating a potentially short half-life. Therefore, the efficacy of lumican can be improved by developing a long-acting lumican derivative using drug-modification technologies.

Skeletal muscle ECM plays an important role in biological reservoir of muscle stem cells and force transmission, maintenance, and repair of muscle fiber [41]. It was reported that lumican regulated ECM assembly and interacted with other ECM components [42]. Thus, lumican may effect on ECM in endo- and perimysium of muscles to exert muscle physiology. In addition, lumican may also act on another organs consisting of striated muscles, such as heart and gastrointestinal tracts, based on the following findings. Integrin receptors [43,44] and lumican [45] ubiquitously distributed throughout the body, including heart and intestines. Favorable effects of exercise on the cardiovascular system have been well described [46], and exercise at right levels may have potential benefits also on the gastrointestinal tracts [47]. Thus, lumican may, as an exerkine, mediate exercise-induced beneficial effects on cardiac and gastrointestinal tracts. These are needed to be investigated.

In conclusion, our in vitro and in vivo experimental results demonstrate the novel role of lumican as a muscle-secreted exerkine that stimulates myogenesis by activating p38 MAPK via integrin receptors. Notably, our results suggest that lumican can be developed as a novel therapeutic agent to afford protection against muscle loss.

## 4. Materials and Methods

### 4.1. Cell Culture

Mouse C2C12 myoblasts were purchased from the American Type Culture Collection (Rockville, MD, USA) and maintained in Dulbecco’s Modified Eagle Medium (DMEM) supplemented with 10% fetal bovine serum (Gibco, Grand Island, NY, USA), 100 U/mL penicillin, and 0.1 mg/mL streptomycin (growth medium, GM) in a humidified atmosphere with 5% CO_2_. On reaching approximately 50% confluence, C2C12 cells were subcultured with trypsin (Gibco).

To examine the effect of lumican (R&D Systems, Minneapolis, MN, USA) on cell viability and proliferation, cells were plated in 96-well plates at a density of 3000 cells/well with GM. After 24 h, the medium was replaced with fresh GM containing 0 or 10 nM lumican. Cell viability and proliferation were examined using a Cell counting kit-8 (Dojindo, Kumamoto, Japan) and Cell Proliferation ELISA (Roche, Mannheim, Germany), respectively, according to the manufacturer’s instructions.

For the migration assay, C2C12 cells were plated onto a type IV collagen-coated filter in 6.5-mm transwell inserts on 24-well plates at 1 × 10^4^ cells. The lower chamber of the well was filled with DMEM containing 0.1% bovine serum albumin (BSA), with or without 10 nM lumican. Then, cultures were incubated for 2 h, and migrated cells were stained with crystal violet and manually counted.

### 4.2. In Vitro Myotube Formation

On reaching 90% confluency, C2C12 cells were incubated in DMEM, supplemented with 2% horse serum (differentiation medium, DM) containing various concentrations of lumican. To block signal transduction pathways, cells were pre-treated with SB203580 (p38 inhibitor, Sigma-Aldrich, St. Louis, MO, USA), TC-I-15 (α2β1 integrin inhibitor, R&D Systems), echistatin (ανβ3 integrin inhibitor, R&D Systems), or integrin α7 neutralizing antibody (Origene, Rockville, MD, USA). After 3 days, the cells were fixed in 4% paraformaldehyde for 10 min, permeabilized in 10 mM sodium citrate buffer containing 0.1% Triton X-100 for 10 min, and blocked with 2% BSA for 1 h. Subsequently, cells were probed overnight with an anti- myosin heavy chain (MyHC) antibody at 4 °C. The cells were incubated with Alexa Fluor 555-conjugated secondary antibody (Invitrogen, Carlsbad, CA, USA) for 1 h, and nuclei were counterstained with 4’,6-diamidino-2-phenylindole (DAPI) for 10 min. Fluorescence images were obtained using an Axio Imager microscope (Carl Zeiss, Oberkochen, Germany) at 100× magnification. Area of MyHC^+^ myotubes and number of nuclei in MyHC^+^ myotubes were quantified in six randomly selected fields per group. Fusion index (%) was calculated as follows: 100 × number of nuclei in myotubes with two or more nuclei/total number of nuclei.

### 4.3. Real-Time PCR and Western Blot Analysis

Total RNA was isolated using TRIzolTM (Invitrogen), and cDNA was synthesized using 2 μg of total RNA with SuperScript^®^ III First-Strand Synthesis System, according to the manufacturer’s protocol (Invitrogen). Real-time PCR was performed on LightCycler 480^®^ (Roche), using SYBR Green I Master Mix. The primer set sequences are provided in Supplementary Table S1.

Total cell lysates were prepared, and western blot analyses were conducted as described previously [32]. Antibodies are listed in Supplementary Table S2. Signals were visualized with Western Lighting Plus-ECL Chemiluminescent Reagents (Perkin Elmer, Waltham, MA, USA), and the intensity of the resulting bands was quantified by ImageJ software (NIH, Bethesda, MD, USA).

### 4.4. Protein Synthesis and Degradation Assays

The rate of protein synthesis was determined using the SUnSET (Surface Sensing of Translation) technique. Confluent C2C12 cells were serum starved for 4 h to induce myoblast differentiation, and the cells were incubated in serum-free media containing 0 or 10 nM lumican for 1 h. Next, the cells were treated with 10 μM puromycin for 30 min, and the incorporation of puromycin into newly synthesized proteins was analyzed by western blotting using anti-puromycin antibody. The rate of protein degradation was calculated from the rate of release of trichloroacetic acid-soluble radioactivity into the medium, as previously described [32].

### 4.5. AAV Cloning and Production

For AAV-based shRNA expression, the control shRNA served as a nontarget control shRNA containing nonhuman or mouse shRNA (5′-TCGCATAGCGTATGCCGTT-3′) and mouse lumican shRNA (5′-CCTGGAAACTCGTTTAATATA-3′) were synthesized using the following method: two kinds of oligos were purchased (Sequence of oligos; [phos]5′-t [sense sequence of target] ttcaagaga [reverse complement sequence of target] ttttttc-3′ and [phos]5′-tcgagaaaaaa [sense sequence of target] tctcttgaa [reverse complement sequence of target] a-3′). The two oligos were annealed with an annealing buffer (in mM; 200 potassium acetate, 60 HEPES-KOH, 4 Mg-acetate, pH 7.3 was adjusted using KOH) and incubated at 95°C for 5 min, followed by 70 °C for 10 min. The annealed double-stranded oligo was inserted into HpaI-XhoI restriction enzyme sites within the pSicoR vector (#11579, Addgene, Watertown, MA, USA) and verified by sequencing. The pSicoR-shRNA vectors with XbaI/HindIII-containing enzymatic site were inserted into the pAAV-minCMV-mCherry (#27970, Addgene) vector in the same enzymatic site. The minimal cytomegalovirus (CMV) promoter in these vectors was replaced with a human CMV immediate-early enhancer and promoter containing b-globin intron by infusion cloning (Clontech, In-Fusion^®^ HD Cloning Kit). Additionally, these vectors were verified by sequencing.

The viral vectors were pseudotyped, with the transgene of interest flanked by inverted terminal repeats of the AAV2 packaged in an AAV-6 capsid. Subsequently, AAV-U6-scrambled RNA-CMV-mCherry and AAV-U6-lumican-shRNA-CMV-mCherry were purified by iodixanol gradient ultracentrifugation by the KIST Virus Facility. The production titers were approximately 1 × 10^13^ genome copies/mL.

### 4.6. Animals and Treatments

All experimental animal protocols were approved by the Institutional Animal Care and Use Committee of the Asan Institute for Lifer Sciences (No. 2018-12-132 and No. 2019-12-107). All mice were acclimatized to specific pathogen-free conditions at the Asan Institute for Life Sciences. Mice were provided access to rodent chow and water ad libitum.

Young mice versus old mice. Eighteen-month-old male C57BL/6 mice (n = 5) were purchased from the Korea Research Institute of Bioscience & Biotechnology (Daejeon, South Korea). Six-month-old male C57BL/6 mice (n = 5) were used as young controls.

Exercised mice versus unloading mice. Eight-week-old male C57BL/6 mice (n = 5) (Orient Bio Inc., Seongnam, Korea) were subjected to exercise with treadmill running at a speed of 12 m/min at a 10° incline for 30 min/day for 4 weeks. In the unloading group, 9-week-old male C57BL/6 mice (n = 5) underwent tail suspension (hindlimb unloading) for 3 weeks. Mice in both groups were sacrificed at 12 weeks of age.

Lumican knockdown by AAV-delivered shRNA. Eight-week-old male C57BL/6 mice were individually placed in a cage with a voluntary wheel running system (Starr Life Sciences Corp, Oakmont, PA, USA). AAV6, including lumican shRNA (AAV6-lumican shRNA, n = 5) or scrambled (AAV6-scrambled, n = 4), were injected into the left GC muscle of mice (once weekly for 4 weeks). Injection titers were 6.5 × 10^10^ genome copies. The same volume of phosphate-buffered saline (PBS) was injected into the right GC muscle.Lumican treatment. Lumican treatment was performed in two mouse models exhibiting muscle loss induced by sex hormone deficiency and disuse, respectively. Sex hormone deficiency was induced by bilateral OVX in female 9-week-old C57BL/6 mice. On reaching 15 weeks of age, mice were injected with PBS (n = 4) or 2 μg lumican (n = 6) through the tail vein once daily for 5 days a week, for 4 weeks in total. Sham-operated female C57BL/6 mice (n = 5) were used as the control. Disuse was induced by hindlimb unloading with tail suspension in male 8-week-old C57BL/6 mice. Then, 5 μg lumican was injected into the left TA muscle of tail-suspended mice twice weekly for 2 weeks. Four mice were used for each group. TA was chosen because it was technically easy to be injected, and because it consists mostly of type 2 fibers in C57BL/6 mice although it is quite heterogeneous muscle [48].

Mice were euthanized by performing cardiac puncture under anesthesia induced by intraperitoneally administering 50 mg/kg tiletamine/zolazepam (Zoletil; Vibrac Laboratories, Carros, France) and 10 mg/kg xylazine hydrochloride (Rompun; Bayer Korea, Seoul, South Korea). The GC, SOL, TA, and EDL were isolated and weighed. Relative muscle mass (%) was expressed as a percentage of body weight.

### 4.7. Haematoxylin and Eosin (H&E) Staining and Immunofluorescence

Muscle tissues of mice were fixed in 4% paraformaldehyde for 2 days, then immersed in 30% sucrose for 2 days, embedded in an optimal cutting temperature compound, and frozen rapidly in liquid nitrogen. Then, 10 μm sections were cut using a cryostat microtome (Leica Microsystems, Wetzlar, Germany) and stained with H&E. The CSA of muscle fiber was determined by manually drawing contours around individual fibers using ZEN 2 (blue edition) software (Carl Zeiss). For immunofluorescence examination, sections were incubated with FITC-labeled wheat germ agglutinin to stain sarcolemma.

### 4.8. Statistical Analysis

Results are expressed as mean ± standard error of the mean (SEM) and provided as individual values within bar graphs. The Kolmogorov-Smirnov test showed the normality of the distribution of all data (*p* = 0.474~0.995). Differences between two groups and among three or more groups were examined by performing an unpaired *t*-test and one-way analysis of variance with Turkey’s multiple comparison test, respectively, using SPSS software, version 21.0 (Chicago, IL, USA). Differences were considered significant at *p* < 0.05.

## Data Availability

Not applicable.

## Supplementary Materials

The following supporting information can be downloaded at: https://www.mdpi.com/article/10.3390/ijms231710031/s1.
