# Supplementary material for: Lumican, an Exerkine, Protects against Skeletal Muscle Loss
Source: Int J Mol Sci. 2022 Sep 2;23(17):10031. doi: 10.3390/ijms231710031 (PMC9456076; doi:10.3390/ijms231710031)
Supplement: Supplementary file 1 [file ijms-23-10031-s001.zip › ijms-1862958-supplementary.pdf]

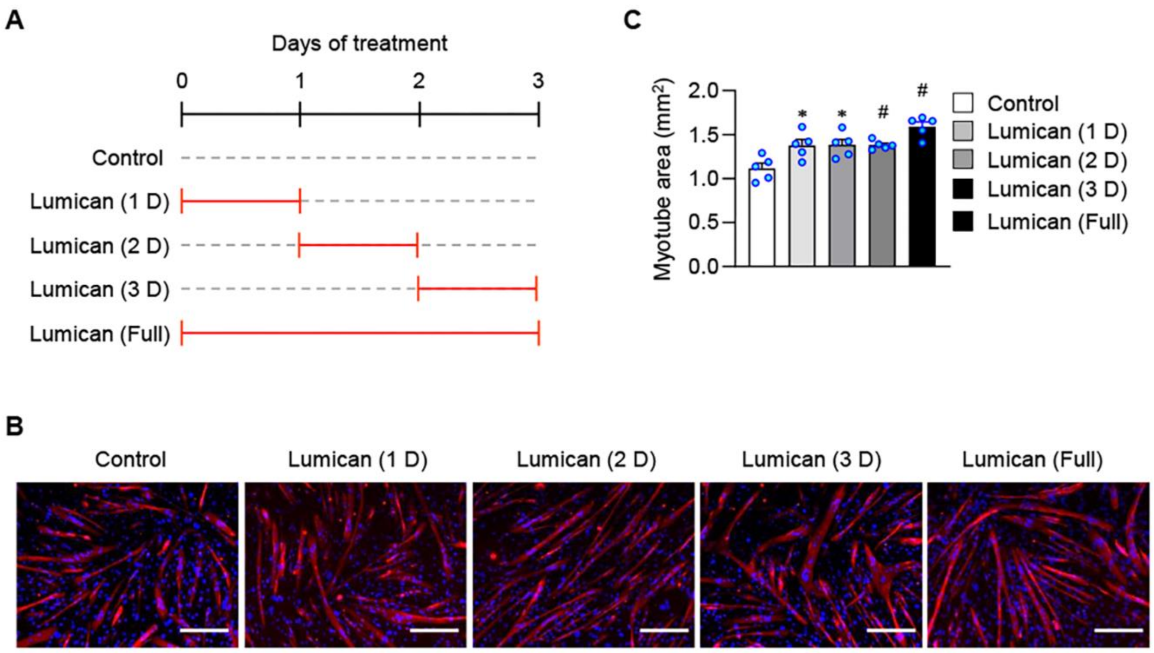

Figure S1. Lumican-mediated myoblast differentiation in cells at various stages of myogenic differentiation. (**A**) Experimental design. C2C12 cells were treated with or without 10 nM lumican for the indicated periods of time. Red lines indicate the periods of lumican treatment. All cells were differentiated for 3 days. (B) Myotubes were stained with the anti-myosin heavy chain antibody. (C) Quantitative results of myotube area per myotube are shown. Each bar represents the mean ± standard error of the mean (SEM). Scale bars: 200 µm. $*P < 0.05$ and $^{\#}P < 0.01$ *vs*. the untreated control.

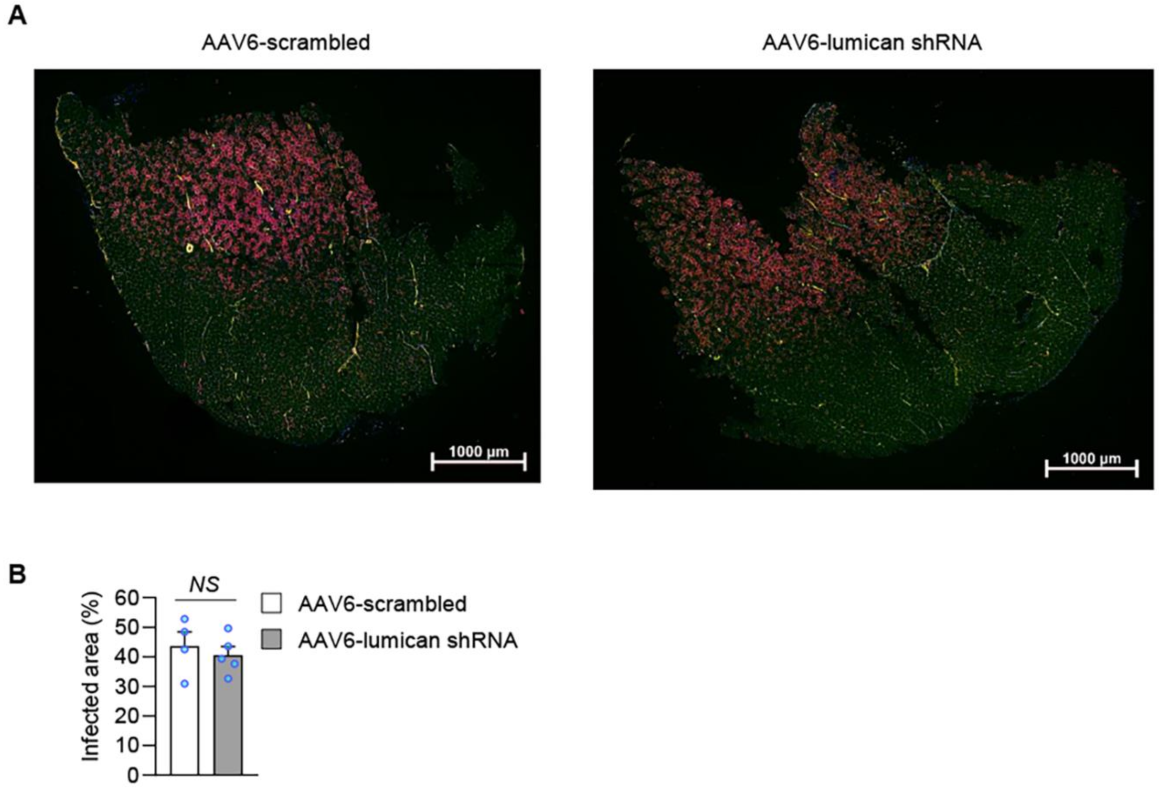

Figure S2. Transduction efficiency of adeno-associated virus serotype 6 (AAV6) in gastrocnemius (GC) muscle. Eight-week-old male C57BL/6 mice were administered AAV6-scrambled-mCherry (n = 4) or AAV6-lumican shRNA-mCherry (n = 5) into the GC muscle (once weekly for 4 weeks). The mice were sacrificed after 4 weeks, and GC muscles were harvested, weighed, and fixed in 4% paraformaldehyde. (**A**) Muscle sections were stained with the FITC-labeled wheat germ agglutinin (green). Red areas represent AAV6-infected areas. (**B**) Percentages of AAV6-infected areas. Each bar represents the mean ± standard error of the mean (SEM). Scale bars: 1,000 µm. *NS*, not significant.

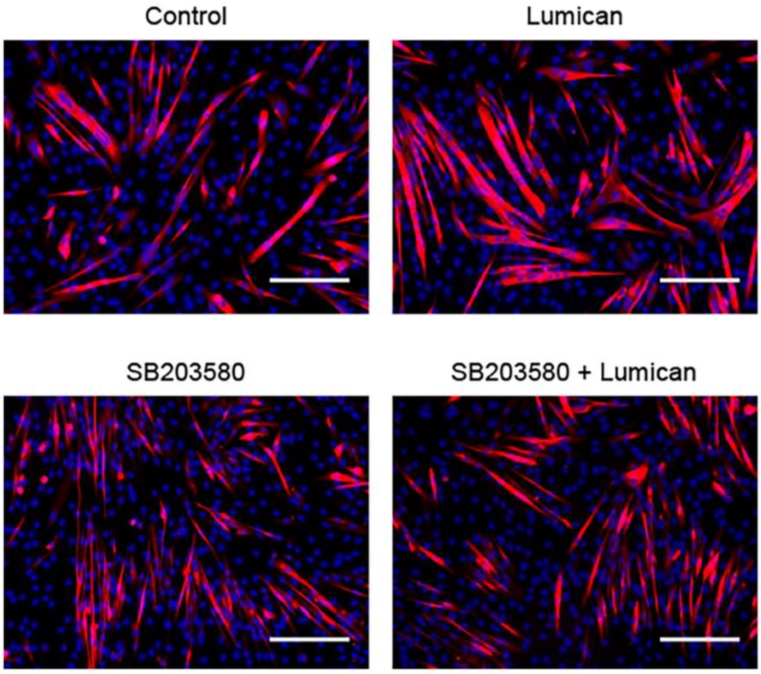

Figure S3. Lumican-stimulated myogenesis mediated by p38 MAPK. C2C12 cells were differentiated with 10 nM lumican in the presence or absence of SB203580 for 3 days. Myotubes were stained with the anti-myosin heavy chain antibody. Representative images are shown. Quantitative results of myotube area per myotube are presented in Figure 5B. Scale bars: 200 μm.

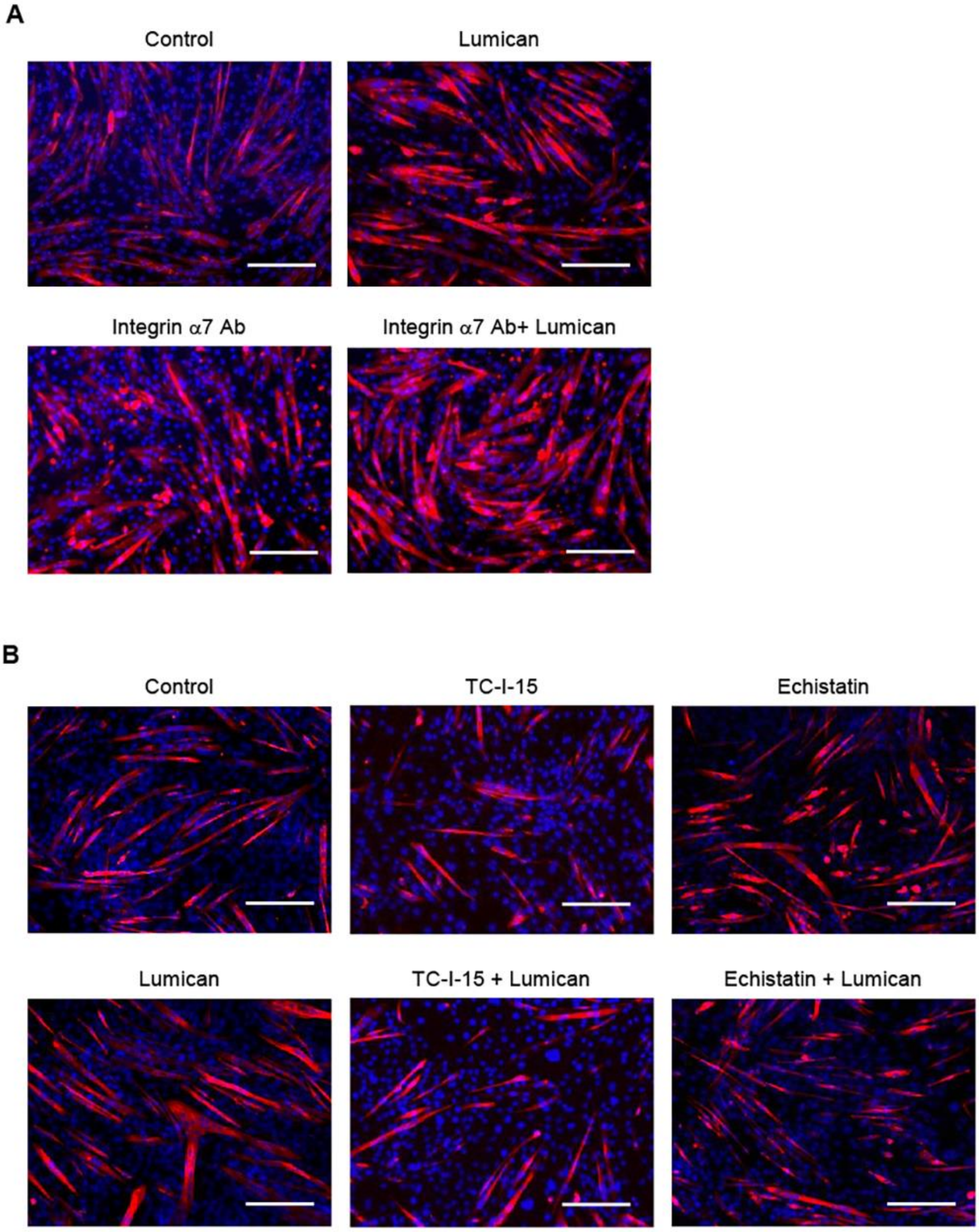

Figure S4. Lumican-stimulated myogenesis mediated by integrins. (**A**) C2C12 cells were differentiated with 10 nM lumican in the presence or absence of integrin α7 neutralizing antibody for 3 days. Myotubes were stained with the anti-myosin heavy chain antibody. Representative images are shown. Quantitative results of myotube area per myotube are presented in Figure 6B. (**B**) C2C12 cells were differentiated with 10 nM lumican in the presence or absence of integrin inhibitors, such as TC-I-15 and echistatin, for 3 days. Representative images are shown. Quantitative results of myotube area per myotube are presented in Figure 6C. Scale bars: 200 μm.

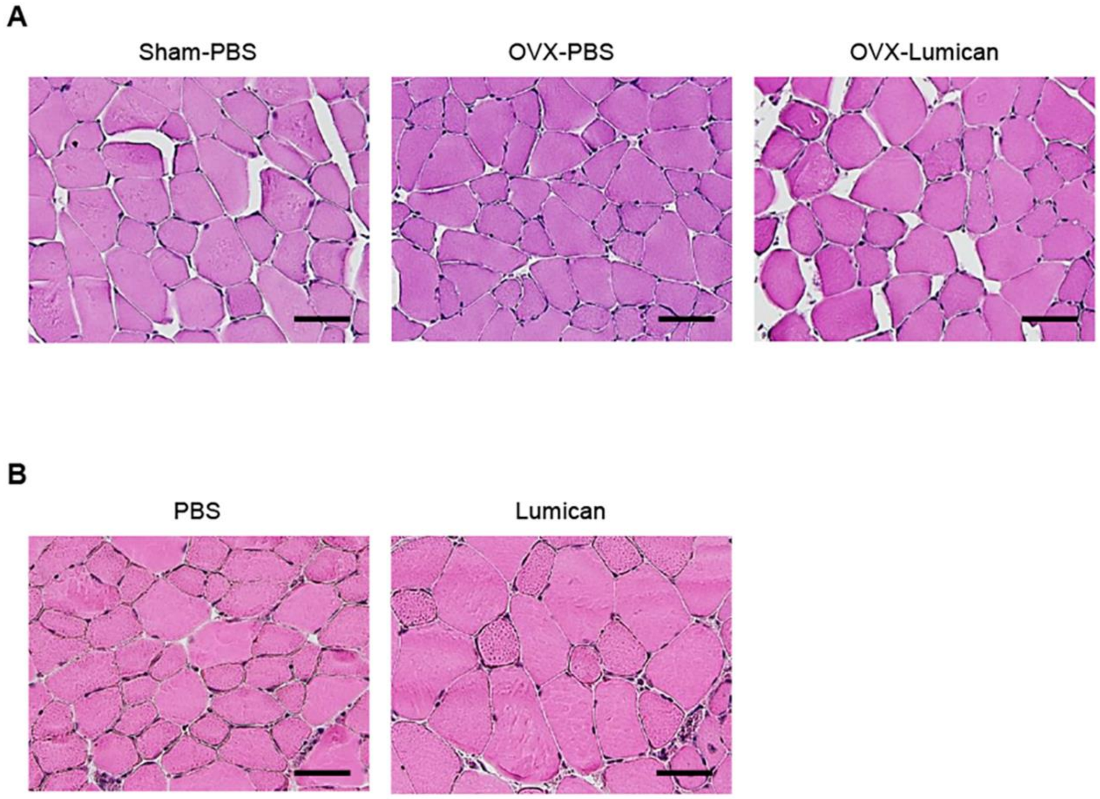

Figure S5. H&E staining in muscles after lumican treatment. (**A**) Ovariectomized (OVX) mice were intravenously injected with PBS or 2 μg lumican for 4 weeks, as described in the Materials and Methods. The mice were sacrificed, and gastrocnemius (GC) muscles were harvested and fixed in 4% paraformaldehyde. GC muscles sections were stained with hematoxylin and eosin. Representative images are shown. Quantitative results of their cross-sectional areas (CSAs) are in Figure 7B. (**B**) Male C57BL/6 mice were subjected to the tail suspension, and lumican (5 μg/mouse) was injected into the left tibialis anterior (TA) muscle twice weekly for 2 weeks. The mice were sacrificed, and TA muscles were harvested and fixed in 4% paraformaldehyde. TA muscle sections were stained with hematoxylin and eosin. Representative images are shown. Quantitative results of their CSAs are in Figure 7E. Scale bars: 50 μm.

Table S1. Primer sequences used for real-time PCR amplification.

mRNA	Primer sequences
18S rRNA	Sense: CTCAACACGGGAAACCTCAC<br>Antisense: CGCTCCACCAACTAAGAACG
Itga2	Sense: GACCAGCTTTTCACCTGCAG<br>Antisense: CAACATCCACGAGGGAAGGG
Itga7	Sense: GATCGTCCGAGCCAACATCACA<br>Antisense: CTAACAGCCCAGCCAGCACT
Itgav	Sense: GGTGTGGATCGAGCTGTCTT<br>Antisense: CAAGGCCAGCATTTACAGTG
Itgb1	Sense: AGTGCTCCCACTTCAATCTCACCA<br>Antisense: TCTCCTTGCAATGGGTCACAGGAT
Itgb3	Sense: CGGGCTAACCGACCAGGTGT<br>Antisense: CCTGCATGATGGCGTCAAAG
Lumican	Sense: CTTTGAGAACGTCACAGACC<br>Antisense:GGACTTTGGAAGTGGACCGA
Mef2a	Sense: CCCAGCCACGCTACATAGAA<br>Antisense: TTGGAGAGGCCCTTGAGTTT
Mrf4	Sense: GACTGCCCAAGGTGGAGATT<br>Antisense: AAATCCGCACCCTCAAGAAT
Myf5	Sense: ACCAGAGACTCCCCAAGGTG<br>Antisense: CAAAGCTGCTGTTCTTTCGG
MyoG	Sense: GCCATCCAGTACATTGAGCG<br>Antisense: GCTGTGGGAGTTGCATTCAC

Table S2. List of antibodies.

List	Company purchased	Catalog number
Anti-actin antibody	Sigma-Aldrich	A3854
Anti-Akt antibody	Cell Signaling Technology	#9272
Anti-Atrogin-1 antibody	Abcam	ab168372
Anti-ERK1/2 antibody	Cell Signaling Technology	#9102
Anti-Integrin α7 antibody	Origene	AM20012AF-N
Anti-JNK antibody	Cell Signaling Technology	#9252
Anti-Murf-1 antibody	ECM Biosciences	MP3401
Anti-myosin (fast) antibody	Sigma-Aldrich	M1570
Anti-p38 antibody	Cell Signaling Technology	#9212
Anti-p70S6K antibody	Cell Signaling Technology	#9202
Anti-P-Akt (S473) antibody	Cell Signaling Technology	#9271
Anti-P-ERK1/2 (T202/Y204) antibody	Cell Signaling Technology	#9101
Anti-P-JNK (T183/Y185) antibody	Cell Signaling Technology	#9251
Anti-P-p38 (T180/Y182) antibody	Cell Signaling Technology	#9211
Anti-P-p70S6K (T389) antibody	Cell Signaling Technology	#9205
Anti-puromycin antibody	EMD Millipore	MABE343
FITC-labeled WGA	Sigma-Aldrich	L4895
